# Nasal Neuroses

**Published:** 1910-06-18

**Authors:** 


### Laryngology and Rhinology.

### NASAL NEUROSES.

Certain vaso-motor disturbances, which are largely neurotic in origin, frequently affect the vascular mucous lining of the nasal passages. *Paroxysmal rhinorrhœa*, also called vaso-motor rhinitis and spasmodic rhinorrhœa, is an affection of this class. It occurs in both sexes among people of a neurotic tendency, and it is frequently induced by mental depression or by some shock or grief. Sexual excitement is an important factor; in many people the vascular mucous membrane over the turbinal bodies becomes erectile under circumstances of sexual excitation, and a permanent condition of turbinal engorgement and nasal obstruction is sometimes the result of masturbation. The symptoms are not constant, but in a typical case recur periodically and consist in fits of sneezing, accompanied by a profuse watery discharge and nasal obstruction. The attacks usually come on in the morning and last for several hours; some patients sneeze thirty or forty times in succession and become greatly exhausted.

### Hay Fever.

*Hay fever* is a very similar condition in which there is present, in addition, a definite source of local irritation. It occurs in neurotic people, generally of the educated classes, and usually shows itself in early adolescence. It is not much seen among hospital out-patients. The irritant is the pollen of many varieties of grasses and flowering plants, and the researches of Dunbar have shown that it is the result not of mere mechanical irritation, but of a definite chemical substance in the pollen. The neurotic predisposition is important, and it is reported that a typical attack of hay fever may be induced by the sight of an artificial rose. In predisposed persons the inhalation of any form of dust may bring on an attack, and some people are affected by driving behind horses or by the presence of a cat. In this country the typical symptoms appear in May or June. The attack begins with itching of the conjunctiva with lachrymation, and this is followed by nasal irritation, paroxysms of sneezing, nasal obstruction, profuse watery discharge, and an extraordinary degree of prostration. Many patients gradually lose their susceptibility with advancing age.

### Signs on Inspection.

On examination of the nose, both in paroxysmal rhinorrhœa and in true hay fever, the mucosa is found to be greatly swollen; the upper and posterior parts are generally pale and present an œdematous, water-logged appearance.

In the treatment of these neuroses the determining factors must be considered. They are the increased reflex irritability of the nervous system, occasionally some abnormality within the nasal passage which renders it unduly sensitive, and, in the case of hay fever, the specific irritant.

Nervine tonics, such as strychnine, arsenic, and valerian, are indicated together with attention to the general health. Change of air is often advisable, and a locality as free as possible from pollen should be chosen for hay-fever patients. Many of these keep well, or but little affected, at the seaside; others appear able to escape the irritation only on the high seas or on a small island; Heligoland has for long enjoyed a good reputation for this purpose. Dunbar has obtained an antitoxin by inoculation of horses with the toxin of the pollen, and this should always be tried as it gives relief in a considerable proportion of cases, though in others it is absolutely ineffective. It is prepared in both solid and liquid form, and may be introduced directly into the nasal passages, but usually is applied to the conjunctivæ, whence it reaches the nose by the lachrymal duct.

### Cocaine Sprays.

The application of cocaine and adrenalin relieves the symptoms for a short time, but this effect is very temporary and is followed by a reaction with increase of the turgescence and aggravation of the symptoms; indeed, in some people without hay fever the use of adrenalin is always followed by sneezing and obstruction. The cocaine habit has often been induced by the use of this drug in hay fever, and it should on no account be prescribed. Usually no abnormality is to be detected in the nose in cases of hay fever and paroxysmal rhinorrhœa except the swollen condition of the mucous membrane, which is the result rather than the cause of the affections.

### Irritation.

But sometimes a definite source of irritation is present, which acts by increasing the sensitiveness of the mucosa, such as a septal spur impinging on the outer wall of the nose, or a polypus or an outgrowth from the turbinal which tickles the septum.

In such cases marked relief or complete cure not uncommonly follows removal of the abnormality, but it must never be forgotten that the results of operative treatment are very uncertain in neurotic patients; they are often "cured" for a time by almost any procedure, but the symptoms are apt to recur as severely as before.

The nasal mucosa, especially on the septum near the anterior end of the middle turbinal body, is often hypersensitive, and the light application of the galvano-cautery to these sensitive areas is frequently followed by marked improvement both in hay fever and paroxysmal rhinorrhœa; this result is never permanent and must be repeated at intervals, but a hay-fever patient will often be enabled to go through the season with comfort after one or two applications of the cautery made just before the hay-fever season. This method is quite painless after careful application of cocaine, and causes very little discomfort afterwards; it is therefore well worth a trial in all such cases.

